# Supplementary material for: Are carbon emissions trading and green financial instruments synergistic? -Comprehensive quantitative research based on content analysis
Source: PLoS One. 2024 Mar 7;19(3):e0298601. doi: 10.1371/journal.pone.0298601 (PMC10919601; doi:10.1371/journal.pone.0298601)
Supplement: S1 Data — (ZIP) [file pone.0298601.s001.zip › Data/地级市Z_F工作报告.zip!/╝¬┴╓/░╫│╟╩╨/└┤╫╘╣1⁄2╓┌║┼í╛┬φ┐╦╩2╛▌═°í┐_2018.docx]

各位代表：

　　现在，我代表市政府，向大会报告政府工作，请予审议，并请市政协各位委员提出意见。

　　一、2017年工作回顾

　　过去的一年是本届政府开局之年，经历诸多大事要事。我们喜迎党的十九大胜利召开，接受环保督察、安全检查、扶贫巡视等政治检验，承担城市改造、脱贫攻坚、项目招商、环境整治等艰巨任务，困难矛盾之多、压力挑战之大前所未有。面对严峻复杂形势和极其繁重的任务，我们在习近平新时代中国特色社会主义思想的正确指引下，在省委、省政府和市委的坚强领导下，在市人大、市政协的监督支持下，按照市六次党代会的部署要求，紧紧围绕建设吉林西部生态经济区，突出抓好生态建设、扶贫开发、经济发展、老城改造“四项重点工作”，接续干成一系列开创性工作，取得一系列标志性成果，推动白城振兴发展迈出坚实步伐。

　　（一）积极应对、攻坚克难，经济发展实现稳中有进。践行新发展理念，有效应对经济下行压力，转型升级步伐加快，主要指标增幅在全省靠前。预计全市地区生产总值完成742亿元，增长6%。地方级财政收入完成41.5亿元，下降1.3%。规上工业增加值完成245亿元，增长4%。固定资产投资完成789亿元，增长7%。农业结构不断优化。减玉米、增水稻、扩大杂粮杂豆种植面积，水田发展到281万亩，成为全省水田第一大市；畜禽养殖标准化水平不断提高，规模养殖达到48%。克服干旱、风雹等自然灾害影响，粮食生产保持稳定。工业经济提质增效。实施投资超亿元的敖东药业、银诺克药业、中一精锻、永固连杆等技改项目51个，50万千瓦光伏领跑者基地项目正式获批。新生成小微企业300户，新升规企业33户，新增高新技术企业、省级科技小巨人企业8户，形成梯次结构。规上工业总产值增长8%、利润增长30%，均居全省前列。服务业攻坚扎实推进。现代物流、生态旅游等产业发展势头良好。电子商务蓬勃兴起，市县乡村四级电子商务服务体系逐步健全。服务业增加值增长8%。民营企业户数增长27%，实体经济不断壮大、活力增强。

　　（二）聚焦精准、招大引强，项目建设实现重大突破。落实全省“三抓”“三早”部署，开展了“三精准项目攻坚年”活动。实施5000万元以上项目257个，其中亿元以上项目171个、10亿元以上项目20个。精准谋划提升层次。遵循“产业树”理念，实行市县联动、部门互动，精准包装超亿元项目143个，做到对上可申报、对外可招商。精准服务优化环境。出台了《关于进一步加强经济发展软环境建设的意见》，实行重点投资项目全程代办制。开展软环境专项整治，组织45家涉软部门向社会公开承诺。集中优势力量帮助招商企业解决难题，为项目建设营造最佳环境、提供最优服务。精准招商成果显著。积极参加“东博会”“吉商大会”，在域内外组织了重点产业项目发布会、燕麦产业项目合作会等5场大规模招商活动，共签约项目80个，签约总额928亿元。投资30亿元的阳春羊奶、投资52亿元的牧原生态养猪、投资65亿元的吉运肉牛等一批三产融合项目开工建设。投资40亿元的大北农现代农业一体化等58个超亿元项目签约，特别是成功引进投资100亿元的梅花集团300万吨玉米深加工项目，改写了白城没有“顶天立地”大项目的历史，将使我市产业结构发生根本性变化。

　　（三）注重保护、加强整治，生态环境得到明显改善。出台生态保护与建设总体规划，启动了生态保护与建设五年行动计划。生态工程扎实推进。新连通水库泡塘64个，河湖连通三年工程提前一年全部连通，蓄水能力达到34亿立方米。蒙草万亩草原修复工程进展顺利，全市规模种植燕麦20万亩，综合治理草原170万亩。持续开展“三年造林还湿双百万”活动，植树造林38万亩，修复湿地56万亩。向海生态移民进入收尾阶段。环境治理力度加大。全面落实生态环境保护工作职责，实现“河长制”全覆盖。因地制宜探索秸秆综合利用，秸秆露天焚烧得到有效治理。整合市区供热企业9家，新增热电联产供热面积430万平方米，实现燃煤小锅炉“清零”。淘汰黄标车完成省下达任务。空气质量优良天数比例达到92.9%，居全省第一位。督察整改效果明显。全力配合中央环保督察，办理信访案件373件，责令整改217家，群众反映强烈的环保问题妥善解决，生态环境持续改善。

　　（四）精准施策、巩固提升，脱贫攻坚取得显著成效。举全市之力，推进精准扶贫、精准脱贫。产业扶贫持续加力。主推“龙头企业+贫困户”“合作社+贫困户”模式，新增产业扶贫项目1113个，带动11.7万人。发展庭院经济10万亩，带动12.7万户，实现贫困村整村推进。发展棚膜13.8万亩，洮北区、大安市棚膜经济形成规模。384个贫困村建设了村级光伏电站，享受到新能源补贴政策，保证收益20年。全面完成6.2万眼“柴改电”。投入监管不断加强。加大扶贫资金整合力度，延产业链金融扶贫模式有效推广，全市投入扶贫资金44亿元，小额扶贫贷款居全省第一。制定扶贫项目和资金监管制度，在全国率先出台农村扶贫资产管理暂行办法，确保资金安全、资产保值、发挥效益。扶贫政策创新叠加。医疗“三下沉两提高一兜底”经验在全省推广，实现看小病不出村、大病全兜底。慢病贫困患者门诊综合报销比例达到85%，大病贫困患者在县域内住院报销比例达到95%，患有28种大病的重度贫困患者住院医疗全免费。启动肿瘤救助政策，对患有32种肿瘤疾病的贫困患者，实施免费住院治疗。出台教育扶贫实施意见，对学前到高中阶段的贫困学生，分类进行减免和资助。制定贫困残疾人帮扶救助政策，切实解决就学、就业、就医等方面实际困难。人居环境明显改善。扎实推进八项工程，全面完成安全饮水和危房改造任务。镇赉县针对D级危房改造户需求，创新实施除险加固模式，得到住建部高度认可。通榆县利用易地扶贫搬迁、城乡建设用地增减挂钩政策，探索形成“陆家模式”，得到国土资源部充分肯定。包保帮扶更加深入。市县乡三级同步启动万名干部驻村，创新开展“三帮扶一推动”活动。落实人大代表、政协委员驻村视察调研提出的168个议案提案，促进脱贫攻坚扎实开展。启动“百企助百村”，开展脱贫攻坚公益活动，引导社会力量参与扶贫，构建“大扶贫”格局。预计134个贫困村退出、5.1万人脱贫，累计减贫15.6万人，贫困发生率从17.9%下降到4.1%，脱贫攻坚取得重大进展。

　　（五）合力攻坚、探索创新，老城改造完成预期目标。围绕建设现代化宜居生态城市，市区上下联动，干部群众共同参与，老城改造取得决定性胜利。完成整体改造。累计拆违18万平方米。投资68亿元，实施16类469项工程，改造主要街路48条，建设地下管廊28公里，改造全部老旧小区172个，新建改造公园、广场6个，实现“三年工程、两年完成”“老城变新城、小区变花园”目标。打造白城样板。抓住试点机遇，充分体现海绵元素，在全国探索出“海绵城市+老城改造”的“白城模式”，得到国家海绵城市建设专家组和住建部的高度肯定，打造了北方寒冷缺水地区海绵城市建设典范。管理同步跟进。理顺城市管理体制，组建城市管理行政执法局。落实《白城市市容和环境卫生管理条例》，实行联合执法，开展专项整治，实施机械化保洁。智慧城市指挥中心竣工，重点区域视频监控全覆盖。市民文明素养普遍提升，获得感、幸福感、自豪感显著增强。这两年是白城城市建设投资最多、规模最大、改造最彻底、变化最明显的时期，中心城市面貌发生了全方位、根本性改观。

　　（六）推进改革、扩大开放，发展动力活力不断增强。始终坚持务实推进改革，全力拓展开放空间。重点改革不断深化。供给侧结构性改革深入推进，“三去一降一补”有效落实，落后产能加快化解，降低企业税费成本5.4亿元。“放管服”改革取得阶段性成效，公布行政审批事项目录，健全事中事后监管制度271项。“多证合一”改革全面实施，32项涉企证照归集为一个执照。农村土地确权基本完成，国有林场主体改革、农垦企业改革、供销社综合改革全面启动。机关公车改革全面完成。公立医院改革全面推开。国有资产管理有效加强。改革试点扎实推进。国家农村金融综合改革试验区加快建设，在全省率先建设农村金融综合服务站，在全国首创产业链“雏鹰贷”。区域性“直购电”试点交易电量3.4亿度，新能源智能微电网示范项目获批，扎鲁特特高压配套工程建成投产。五个县（市、区）全部列入粮改饲试点。开放合作深度拓展。推进与长春市、嘉兴市对口合作，全面开展交流对接。承办国际农博会白城农机分会，成功举办中国农业机械（北方）“田间日”活动，组织开展首届科尔沁草原道地关药发展论坛。新成立外埠白城商会4家，畅通交流合作渠道。改善对外交通，长白快铁、白阿铁路电气化改造提前一年通车运营，嫩江大桥主体竣工，白城机场正式通航，对外开放立体交通更加便捷通畅。

　　（七）加大投入、补长短板，民生福祉有了新的提高。在财力趋紧的情况下，重点民生投入达到地方财政支出的81%。民生实事全面落实。在全省率先出台鼓励大学生创新创业、农民工等人员返乡创业优惠政策，已有25户企业享受到市财政补贴奖励。城镇新增就业4.4万人，农村劳动力转移就业27.8万人（次）。城乡居民收入均增长6%。开展老年人意外伤害保险工作，社会保险待遇、最低生活保障和困难群体救助标准进一步提高，与32个省市实现跨省异地就医直接结算。改造棚户区9290套，分配公租房2821套。社会事业协调发展。创新驱动战略深入推进，获得省级科技发展项目22项。推动教育事业均衡发展，新建标准化幼儿园6所，维修改造校舍9万平方米，市中职实训基地投入使用。深化产教融合，开创校企合作良好局面。文化惠民工程全面推进，开工建设滑冰馆，新建贫困村综合文化服务中心147个，扩建农村文化小广场504个。医养结合试点稳步开展。“七五”普法全面启动。“白城好人•引领风尚”等精神文明创建活动蓬勃开展，选树市级以上好人137名。“全民志愿•公益白城”获全省最佳公益服务项目。支持服务国防和军队改革，军人、军属和退役人员权益保障工作有效落实。社会大局和谐稳定。圆满完成党的十九大等重要会事期间信访维稳工作。治安防控全面加强，重大案件及时侦破。开展安全生产大检查、大督察，及时整改安全隐患，未发生较大以上和有影响的安全事故。继续保持重大食品、药品安全事件“双无”目标。民族宗教、外事侨务、广播电视、人口计生、妇女儿童、残疾人、老龄等工作取得新成绩，气象、人防、地震、档案、地方志、红十字会等事业实现新发展。

　　（八）从严治政、转变职能，自身建设全面改进加强。落实全面从严治党要求，政府党组认真履行主体责任。强化政治建设。认真学习宣传贯彻党的十九大精神，深入推进“两学一做”学习教育常态化制度化，扎实开展“解放思想事上见、撸起袖子加油干”主题教育实践活动，“四个意识”“四个自信”进一步增强。强化依法行政。制定了新一届政府党组议事决策规则、政府工作规则等系列规章制度。贯彻法治政府建设实施纲要，依法行政深入推进。重大决策前，全部实行合法性审查。对撤地设市以来的规范性文件进行了全面清理。落实市委关于加强人大工作和人民政协协商民主建设两个意见，自觉接受监督，认真办理人大代表建议、政协委员提案，办复率达到100%。强化纪律作风。严格落实中央“八项规定”精神，驰而不息纠正“四风”。主动认领中央巡视“回头看”反馈问题，扎实推进整改落实。“三公”经费再下降，机构编制“零增长”。严格督查追责，加强行政监察和审计监督，确保政令畅通。落实“一岗双责”，推进政府系统党风廉政建设，反腐倡廉成效明显，政治生态风清气正。

　　各位代表！

　　一年来，我们面对新形势新挑战，迎难而上、加压奋进，取得的成绩实属不易。这是我们在以习近平同志为核心的党中央坚强领导下，全面落实省委、省政府和市委正确决策部署的结果，是人大、政协监督支持的结果，是广大干部群众共同努力的结果。在此，我代表白城市人民政府，向全市各族人民，向各民主党派、工商联、人民团体、无党派人士、离退休老同志，向驻白中省直部门和企事业单位、人民解放军指战员、武警部队官兵、公安司法干警，向所有关心支持白城发展的各界朋友，致以崇高的敬意和衷心的感谢！

　　审视政府工作，我们清醒的认识到，还有许多矛盾需要化解，还有一些工作没有做到位。主要表现在：经济总量不大、效益不高、竞争力不强的问题仍然突出，大部分产业处于产业链低端，资源挖掘不充分，动能转换不够快，推动高质量发展任重道远；生态环境相对脆弱，水、草、林、湿综合治理还有很多欠账，大气、水体、土壤污染治理还需要加大力度，生态环境与群众美好期盼仍有较大差距；精准脱贫的任务艰巨繁重，帮扶工作还不均衡，产业支撑力、企业带动力还不够强，政策宣传落实还不够好，群众脱贫内生动力、稳定增收能力有待进一步增强；城市承载功能和整体品位亟待提升，公共设施不配套、不完善，管理维护不到位、不精细，打造绿色、智慧、人文的新型城市还有许多工作要做；民生社会事业发展存在短板，在就业、教育、医疗、养老、住房、食品安全等方面，还有群众不满意的地方，有的诉求没有得到很好解决；软环境建设亟待加强，市场准入、项目立项、行政审批等环节不够优化，融资难、成本高、效率低的现象仍然存在，投资、营商、人才等环境还需进一步改善；政风建设有待改进，有的干部不作为、不敢为、不会为，工作招法不多，责任担当不够，建设勤廉政府还需持续发力。问题是时代的声音，也是今后的努力方向。我们一定直面问题、勇于担当，全力以赴做好政府工作，不辱历史使命，不负人民重托。

　　二、2018年工作安排

　　党的十九大作出了中国特色社会主义进入新时代的重大论断，确立了习近平新时代中国特色社会主义思想，描绘了全面建设社会主义现代化国家的宏伟蓝图。围绕学习贯彻党的十九大精神，省委十一届二次全会对决胜全面小康、建设幸福美好吉林进行了战略安排。市委六届四次全会紧扣让全市人民过上更加美好的日子，精心谋划部署，开启了新时代白城全面振兴发展的新征程。

　　今年，是全面贯彻党的十九大精神的开局之年，是决胜全面建成小康社会、实施“十三五”规划承上启下的关键一年。政府工作总的要求是：深入学习贯彻党的十九大精神，高举习近平新时代中国特色社会主义思想伟大旗帜，全面落实省委十一届二次全会、市委六届四次全会部署要求，坚持稳中求进工作总基调，按照高质量发展的要求，统筹推进“五位一体”总体布局、协调推进“四个全面”战略布局，大力实施“三个五”发展战略，加快建设吉林西部生态经济区，打好防范化解重大风险、精准脱贫、污染防治三大攻坚战，抓好生态环境、生态扶贫、生态经济、生态城市“四项重点工作”，统筹推进稳增长、促改革、调结构、惠民生、防风险各项工作，不忘初心、牢记使命，努力为全面建成小康社会、让全市人民过上更加美好的日子不懈奋斗。

　　坚持以高质量发展为前提，加快发展速度，注重结构优化，着力扩大总量。主要预期目标是：地区生产总值增长6.5%左右；地方级财政收入增长6%左右；固定资产投资增长7%左右；规上工业增加值增长7%左右；社会消费品零售总额增长5%以上；居民消费价格指数涨幅控制在3%以内；城乡居民收入与经济基本同步增长；城镇新增就业3.8万人，登记失业率控制在4.5%以内；节能减排指标完成省下达任务。

　　重点抓好以下十个方面工作：

　　（一）抓好生态建设，打造美丽白城。深入实施生态保护与建设五年行动计划，扎实开展治理保护攻坚战。一是实施生态建设工程。推动河湖连通达标提升，完善64个骨干工程配套设施，及时蓄水发挥效益；科学谋划地下水库、区域水循环系统工程，加快破解“水”瓶颈。推进草原治理工程，推广“劲草一号”2万亩，种植燕麦30万亩，加快草原生态观光一体化，把草原打造成为白城的生态名片。推进造林绿化工程，突出绿色大通道、滨水景观带、环城绿化带建设，完成造林37万亩。推进湿地修复工程，抓好引水增湿、退耕还湿、芦苇修湿，修复湿地24万亩，全面完成“三年造林还湿双百万”任务。二是加强环境污染防治。启动污染治理攻坚行动，实施蓝天工程，全面完成集中供热整合，新增电能清洁供暖150万平方米，抓好秸秆禁烧和能源化、饲料化、原料化利用；实施清水工程，全面落实“河长制”，设立“湖长制”，加强流域治理、污水处理、水源地保护，开展河道清洁整治，确保水环境质量安全；实施净土工程，加强农业面源污染防治，开展农药化肥零增长行动，完成禁养区内畜禽养殖小区搬迁，治理水土流失20万亩。严格实施“禁塑令”，倡导绿色消费。三是完善生态建设和环境保护制度。抓好中央环保督察反馈问题整改落实，建立市级环保督察机制。严格环境准入，坚守生态红线。落实草原禁牧、草畜平衡制度，加快制定生态领域地方性法规，健全生态建设制度体系和生态保护法治体系。依法打击破坏环境违法行为，保护好绿水青山、碧草蓝天。

　　（二）抓好脱贫攻坚，扩大扶贫成效。坚决打好产业扶贫、基础设施建设、双扶双带、政策落地“四场硬仗”，确保真脱贫、脱真贫。一是强化产业扶贫。实施产业、创业、就业“三业联动”，推广“龙头企业+贫困户”“合作社+贫困户”带动模式，引导规上企业与双带项目相融合，鼓励企业建立“扶贫车间”。大力发展庭院经济，全面落实补贴和订单，实现所有行政村全覆盖。制定完善项目带户利益分配制度。深入落实大学生和返乡农民工创业扶持政策，抓好守家经济、电商扶贫，打造农村半小时就业服务圈。二是强化人居环境改善。加快实施八项工程，全面完成村屯道路、绿化美化、村卫生室、文化活动室等建设任务，推动农村基础设施建设提档升级。利用耕地占补平衡、城乡建设用地增减挂钩指标交易政策，盘活土地资源，再造耕地、恢复生态，保障投入、反哺“三农”。开展农村人居环境整治行动，搞好垃圾处理，推进“厕所革命”，建设美丽宜居乡村。三是强化包保帮扶。注重扶贫与扶志、扶智相结合，全面开展“大培训”活动，抓好“三帮扶一推动”“百企助百村”等工作，实施科技扶贫“十百千万”工程。搭建社会扶贫信息服务平台，完善企业、商会等社会力量参与机制，持续推进“公益白城”“圆梦助学”“日行一善”等扶贫公益项目。四是强化政策落地。全面落实“三下沉两提高一兜底”、教育扶贫等政策，推进“健康快车”项目，探索困难救助特事特办、一事一议新举措，落实残疾人、精神病患者、失能老人救助等政策。整合涉农资金，强化金融扶贫，扩大小额贷款投放领域。对照国检标准，加强自查整改。严格督查考核，强化结果运用。开展“脱贫攻坚作风建设年”活动，搞好扶贫领域作风问题专项整治。全市要确保3.5万人脱贫、217个贫困村退出，完成4个县（市、区）摘帽，努力让群众认可、经得起检验。

　　（三）抓好项目建设，强化投资拉动。深入落实“三抓”“三早”要求，着力开展“三大行动”，以项目突破带动转型升级，以优质增量加快动能转换。一是开展大项目攻坚行动。计划实施超5000万元的重点项目200个以上，重点抓好梅花玉米深加工、聚能重工综合板块、鲁能风电基地等超百亿元项目开工建设。各县（市、区）、市直工业园区和开发区要至少新建2个投资超10亿元的工业项目。二是开展点对点招商行动。围绕打造全产业链，聘请专家智库论证，深度谋划超亿元项目100个、超百亿元项目10个。实施“一把手”招商工程，举办“鹤商大会”“特色产业推介会”等活动，加强与行业领军企业联系，努力在重大项目引进上实现更大突破。三是开展软环境提升行动。整合优化政务服务工作流程，实施企业投资项目审批流程再造。积极构建“亲”“清”新型政商关系，制定政务诚信清单，认真履行招商引资承诺，着力打造“诚信白城”。突出涉软案件查办，坚决打通一切“中梗阻”，实现“服务大提升、建设零干扰、发展无障碍”。

　　（四）抓好工业转型，增强支撑能力。以深入推进工业转型升级三年行动为载体，构建产业发展、企业成长、创新驱动、要素保障、政策支撑“五大体系”，力争规上工业总产值突破900亿元。一是深入推进小微企业生成计划。鼓励支持大众创业、万众创新，实施百个重点创业项目、百户重点小微企业升规工程。加快7个“双创”中心建设，搭建小微企业生成平台。落实科技企业孵化器税收减免、大学生创业补助奖励等政策，吸引高科技人才，推动实现低成本创业。新发展小微企业300户以上，新升规企业40户，民营企业户数达到1.5万户。二是深入推进重点企业培育计划。实施百户重点企业成长工程，产值超亿元企业达到184户、超10亿元达到4户。实施百个重点工业项目，全力支持重通成飞、长恒药业等重点企业改造升级，技改项目投资达到135亿元，占工业投资的60%。持续开展“四包一联”，推动停产半停产企业复工增产，保证重点企业稳定生产。三是深入推进重点产业跃升计划。围绕做大做强“两支两优”产业，实施百个重点创新项目，集中打造清洁能源、燕麦产业、医药健康、绿色锻造、农产品加工等百亿级园区。探索“光伏+”模式，完成50万千瓦光伏领跑者基地建设。重点抓好阳春羊奶150万只奶山羊、吉运10万头肉牛等全产业链项目。推动建龙集团30万吨绿色铸锻、国药集团中药提取颗粒等重大产业项目建设。强化工业对县域经济的支撑作用，推进开发区（园区）创新转型、错位发展。实施企业家素质提升工程，激发和保护企业家精神，全面释放创新创造活
